# Reviews of Books

**Published:** 1923-07

**Authors:** 


### REVIEWS OF BOOKS.

### PATHOLOGY IN HISTORY.

**Post-Mortem: Essays, Historical and Medical.** By C. MacLaurin, M.B.C.M., F.R.C.S.E., LL.D. (Cape; 7s. 6d. net.)

Dr. MacLaurin is Lecturer in Clinical Surgery in the University of Sydney, and he has applied his medical knowledge and his pathological curiosity to an inquiry into the diseases and physical abnormalities of great historical personages. Someone else recently wrote a book in the same order of ideas about Queen Elizabeth, and the prospect thus opened up of a small library devoted to the diseases of the famous is a trifle alarming. Yet the curious interest of such books is undeniable, and it is obviously impossible to restrict these inquiries within professional circles. Dr. MacLaurin is a lively and attractive writer, notwithstanding the gruesome material in which he works, but he is likely to alienate many readers by his unnecessary and offensive gibes at religion. That is not the right temper of the historian, and, although he makes no pretence to original research, he is well read in history and forms conclusions which would seem, in the main, to be just and reasonable.

Dr. MacLaurin's patients, if we may make this use of the word in relation to the eleven famous personages whose cases he considers, were not all abnormal. The malady of Samuel Pepys was common then, and is common now, and, although there may have been abnormalities about Napoleon, the cancer of the stomach which killed him was not among them. That his disease was wrongly diagnosed and that Antomarchi refused almost to the end to believe that there was much the matter is notorious. For the rest, Dr. MacLaurin's verdicts, if they may be accepted—and around these subjects controversy will rage to the end of time—suggest that historians might do well to call in the doctor before they finally sum up the great or the notorious characters who form landmarks in history. Thus, it would seem, Anne Boleyn was a nymphomaniac; Joan of Arc never became a woman; Mary Tudor suffered from a "supressed sex-complex"; Charles V. died of chronic Bright's disease, and his son, Don John of Austria, the victor of Leparto, of ruptured typhoid ulcer; Gibbon of rupture and a hydrocele; while Benvenuto Cellini, the author thinks, was syphilitic. It is rather a terrible catalogue, and it is fairly certain that some at least of these tragedies might have been averted, and on this point Dr. MacLaurin has some interesting speculations. After dismissing the suggestion that that remarkable woman, the Empress Theodora, wife of the equally remarkable Justinian, suffered from the minor venereal affection, he writes :—

"A more probable explanation of her continued ill-health might be that she became septic at her confinement, when the unwanted girl was born. When the Byzantines spoke of a child as being 'born in the purple,' they spoke literally, for the Roman Empress was always sent to a 'porphyry palace' on the Bosphorus for her confinement, and once there she had access to less good treatment than is available for any sempstress to-day. It is impossible to suppose that the porphyry palace—the 'purple house'—ever became infected with puerperal sepsis, because there was never more than one confinement going on at a time within its walls, and that only at long intervals. Still, there must have been a great many septic confinements and unrecorded female misery from their results among the women of the early world; and that must be remembered when we consider the extraordinary small birth rate of the Imperial families during so many centuries. Had the Roman Emperors been able to point to strong sons to inherit their glories, possibly the history of the Empire would have been less turbulent. A Greek or Roman Lister might have altered the history of the world by giving security of succession to the Imperial despot."

Had Napoleon remained in bodily vigour for a few years longer, he might have escaped from St. Helena, and made one more bid for dominion—for that matter, he might even not have lost Waterloo, "the nearest run thing you ever saw," as Wellington said to Creevy a few days afterwards. But the might-have-beens of history are the most fascinating of all "Ifs," and the least satisfying. We can only play with history; those who made it played with the destinies of mankind.

Having ranged from a "minx" like Anne Boleyn, and a mystery like the Maid of Orléans, to a glutton like Charles Quint, and a delusionist like Marat, Dr. MacLaurin concludes with an essay on Death which, clever and suggestive as it is, will not give comfort to everyone. He finds nothing terrible in the act of death—most men, indeed, in his experience, are unconscious hours, or days, before they die. It is only, he thinks, the old who fear death—the longer we live the more strenuously we cling to life. Nothing is more tragic than the death of "the young, the brave, the fair"; yet the tragedy is for us rather than for them. And as they look out from the gold bar of Heaven they may marvel at our tears and pity us for our poverty of understanding.

---

### "NAUHEIM" IN THE HOME.

**The "Nauheim" Treatment of Diseases of the Heart and Vessels in England.** By Leslie Thorne Thorne, M.D., B.S.(Durham), M.R.C.S.(Eng.), L.R.C.P.(Lond.) (Baillière, Tindall & Cox; 7s. 6d. net.)

The author, who has practised the "Nauheim" treatment for over 20 years in London, believes that this treatment can be given in any place where there is a bathroom, and a good supply of hot water. Hence his book, which gives a clear account of how to apply the treatment at home. In his opinion it is within the power of every medical man who will take the necessary trouble to carry it out there with results as good as, if not better than, are obtained at Nauheim, "where it is given in that wholesale manner common to spa treatment, and where the large number of patients makes it practically impossible for the physician to supervise the bathing in each individual case as carefully as he should do if he wishes to get the best results." The author's account is full and lucid, and the many illustrations are useful in showing how the various movements are to be carried out. The book also serves a useful purpose in correcting common misconceptions with regard to what actually constitutes "Nauheim" treatment. It should prove of value to physicians whose patients suffering from chronic cardiac disease are financially or physically unfit to undertake a long Continental journey.

It is, however, doubtful if the author is well advised in his sweeping and wholesale preference for home treatment. The influences at work in Nauheim are many and varied. There is, for example, the psychic influence—an "atmosphere," the value of which cannot be even approximately gauged. There is also the pecuniary factor; the patient who has temporarily sacrificed his business connections and whose visit to Nauheim has cost him some hundreds of pounds, is much more likely to follow out the treatment conscientiously than the patient who remains at home and just "steps over the way" to be treated. As for the supervision of patients in an institution like Nauheim, it can be made remarkably effective even when the numbers are great. There are, however, many patients whose choice is limited to home treatment or no treatment, and for this class the author's book should prove a great boon.

---

### THE EXPECTANT MOTHER.

**Getting Ready to be a Mother** By Carolyn Conant van Blarcom, R.N., Author of "Obstetrical Nursing," &c., formerly Assistant Superintendent and Instructor in Obstetrical Nursing and Care of Infants and Children at the Johns Hopkins Hospital Training School for Nurses (Macmillan; 6s. net.)

This little book, with its attractive title, contains, as its name implies, "information and advice for the young woman who is looking forward to motherhood," and is dedicated to "the baby, upon whose well-being depends the future race." It is written in a pleasant, easy and conversational manner, and will appeal, not only to those poorer women who attend pre-natal clinics at the infant welfare centres, but to others who have had better educational advantages. There is nothing to alarm and much to encourage the young mother in her study of these eleven simply-written chapters of sound practical advice. While certain dangers and eventualities commonly associated with child-bearing are not ignored or minimised, the reader is continually shown how they can usually be avoided by common sense and a wide knowledge of their causes and prevention.

Information, complete though brief, is given on every subject pertaining to pregnancy, confinement, and the care of the new-born infant that the expectant mother ought to know. Helpful diagrams and illustrations lighten the pages of necessary anatomical description, explain certain "bed-exercises" which it is desirable to take after a confinement, and show the proper methods of feeding, bathing and dressing the baby. An easily understood diagram of the milk-teeth, giving the ages at which they usually are erupted, should be very useful. Two special points are kept prominently before the reader throughout the book—the necessity of skilled medical supervision from the very beginning of pregnancy, and the extreme importance to the infant of breast feeding. An introduction by two doctors, J. Clifton Edgar, M.D., and Frederick W. Price, M.D., adds to the value of the book.

### THE SURGICAL FIELD.

**Urgent Surgery.** By Félix Lejars, Professeur Agrégé à la Faculté de Médicine de Paris. Third English edition, translated from the eighth Fernch edition by W. S. Dickie, F.R.C.S., and Ernest Ward, M.A., M.D., F.R.C.S. 20 full-page plates and 1,086 illustrations. (John Wright & Sons, Ltd., Bristol; 63s. net.)

The title of this truly excellent work must not be taken to imply merely the surgery of injuries, urgent though that may be. *Chirurgie d'Urgence* has as wide an application as the enormous number of clinical conditions nowadays met with by the general practitioner as much as by the casualty officer, all of which may demand immediate surgical action. It is not that the conditions themselves have changed, but that means of surgically combating them have steadily extended. As the author remarks, we are better equipped than formerly, and our responsibilities have thus enormously increased.

Therefore in the present edition we find chapter after chapter describing one by one in a concise yet remarkably complete way, every detail of all the surgical crises a practitioner is likely to meet. Even though he does not operate himself, it is well that the physician should have a clear idea of the possibilities and limitations of modern surgery, and such a book at this provides just what he needs. Also, the sternest lessons of war have here been applied to the surgery of peace, and this is particularly well seen in the sections dealing with visceral injuries and extensive wounds. The plates are really excellent, and the whole production of the book leaves nothing to be desired.

---

### A NEW MEANING TO DREAMS.

**Conflict and Dreams.** By W. H. R. Rivers, LL.D., F.R.S. (Kegan Paul; 12/6 net.)

This is an amazingly interesting book, and the pity is the late Dr. Rivers was not spared to follow out the lines of thought that he here suggests. Accepting the fundamental principles of Freud, that dreams have a serious meaning, he does not regard them as utterly purposeless as Freud did, but urges strongly that the result is not non-creative and useless. Freud, in this, unlike most psychologists, held that dreams were not a mere result of half-faded images of memory, but that each arose causally from unconscious tendencies in much the same way as the thoughts and actions of day-time life—in short, that dreams arise from active workings of some subconscious but very active mentality. Freud's in some ways disappointing conclusion was that a dream is simply a distorted picture prompted by some primitive subconscious tendency, which, though it may reveal this tendency and be therefore helpful in analysing the abnormal mind, has no constructive value, and is no help to the individual in his daily life.

Now Dr. Rivers would have us agree as to the presence of definite subconscious promptings from our primitive selves. From careful analysis of his own experiences, he insists that dreams are directly related to the thoughts and actions of our conscious life. In dreams he detects a definitely constructive process, which, though possibly fantastic in form, directly aims at the solution of problems which confront the dreamer in his waking hours. Though often weirdly expressed, the conclusions reached in our dreams may be better balanced, wiser shall we say, than those of full consciousness.

Why should this be ? The explanation here given is straightforward enough. The natural impulses, the results of training, heredity and so forth, are, in sleep, not automatically set in conflict. Each can play its part unhindered. Imagination can develop its ideals to the fullest. The conscious self is hemmed in by the rules and conventions of daily life. The subconscious self can debate a problem without restraint of any kind. Therefore it would seem that we are to put a totally different interpretation upon our dreams. In them there is little or nothing from which to foretell the future; only partially do they represent the details of the primitive or infantile intelligence upon which our developed mind is built. Instead the main import of dreams may be their simple illustration of how an unbiased intelligence would tackle the unsolved problems of our everyday life. The author develops his theory from his own experiences. It is a more than fascinating exercise to interpret, or at least attempt to interpret. our own dreams in this way. It is certainly pleasanter than the Freudian method ; possibly it is also more true to fact.

### IN THE EYE HOSPITAL.

**Nursing of Diseases of the Eye.** A Simple Treatise for Nurses. By JESSIE ELMS, A.R.R.C., Matron, The Sussex Eye Hospital, Brighton. (The Scientific Press; price 2s. 8d., post free. Illus.)

Probationers in eye hospitals, also private nurses grown a little " rusty," will alike find this book useful and practical. As matron herself of an eye hospital, Miss Elms has had ample opportunity of putting into practice all the theories she teaches, and her book is evidently written from the standpoint of one who has been both a learner and a teacher of others. The little volume contains only eight chapters, but in them the essentials of eye treatment are happily grouped. Chapters one and two deal briefly with the structure and the diseases of the eye, followed by two more on the general nursing treatment and methods employed. Theatre work has a chapter to itself.

The author impresses on her readers the extreme importance of nursing care in eye cases. She says : " Never put in a drop without first looking to see that you have the right medicament and the right strength ordered. An eye may be blinded by a wrong drop being put in, from which you will realise the importance of this point." This is very true, but it would also have been well not only to show illustrations of the correct kind of drop-bottles in use at eye hospitals, but to have added a word of caution as to the dangers of certain popular glass eye-droppers supplied with rubber teats, which unless boiled each time before being used, serve to collect dust likely to be transferred to the lotion used and the eyes treated. The importance, too, of keeping the patient lying on his back in one position for some hours after any serious eye operation is, perhaps, hardly sufficiently emphasised; in other respects, the principles of good eye nursing are clearly demonstrated. All the necessary instruments used during eye operations are well illustrated, and their names may be readily learned from the illustrated lists at the end of the book.

### MISCELLANEOUS NOTICES.

### The Nursing of Infectious Diseases.

LECTURES UPON THE NURSING OF INFECTIOUS DISEASES. By F. J. Woollacott, M.D., D.P.H. (The Scientific Press. 4s. net.)—This is the third edition of a practical little book based on a series of lectures originally published in the nursing section of our own pages. It has been revised and considerably enlarged by Dr. Dorothy Hare, who has added chapters on cerebro-spinal fever, tuberculosis, and venereal diseases.

### Anatomy for Nurses.

TEXT-BOOK OF ANATOMY AND PHYSIOLOGY. By Elizabeth R. Bundy, M.D. Fifth Edition. 1923. J. & A. Churchill. 11s. 6d. net.—In this latest edition of Dr. Bundy's well-known manual the revisions and additions have been made by Dr. Martha Tracy and Miss Grace Watson. These consist mainly of a more detailed description of the ductless glands and of the processes of metabolism, including an account of vitamines. Many little practical notes of a clinical character are appended which add materially to the usefulness of this text-book to nurses in training. The illustrations are always helpful, and the glossary of technical terms is sufficiently full.

### A Nurse's Experiences.

THE ADVENTURES OF A PRIVATE NURSE. By Eva Riddock. Scientific Press. 3s. 6d. net.—These little experiences are told in a vivid and sprightly manner which makes good reading. The experiences of any nurse must be almost as full of humour as of pathos, and many of Miss Riddock's adventures are distinctly amusing. One or two, such as " A Case of Alcohol " or " Should the Doctor Tell ? "—the latter a really terrible little story—illustrate forcibly the dangers and difficulties that sometimes await the nurse, even in circumstances which at first appear simple. There is one story of an appalling nursing home which, though admirable in appearance, was a perfect death-trap for the poor patient who entered it. The place was only partially equipped; although there was a good operating-theatre to impress the operating-surgeon, the nursing staff was totally inadequate. As Miss Riddock points out, it is imperative that " every nursing home should be required to conform to some registered standard and be subject to independent and intelligent supervision."

### A Tale of a Red Herring.

DRAPED IDOLS. By Lilian Arnold. Long. 7s.—Mrs. Arnold enjoys the uncommon possession among contemporary novelists of a sense of literature. If that sense is more or less elusive, it pleases the reader only the more, and she has here added to her former promising work a little study in human character which is at once penetrating and reticent. She deals, not with that " Eternal Triangle " which has grown wearisome by repetition, but with another triangle composed of three women with a man poised perilously upon its apex. He is not the highest type of hero, although there is much to be said for him ; the real characters are the women who are drawn with the quiet vividness which turns a few strokes into a sure and certain portrait. There is a subtle little plot in the book, a plot of freshness and ingenuity, but of the commonplace passion of the novelist there is nothing. It is, in fact, a study in motives—feminine motives, of course. Men merely blunder into situations which women carefully plan, usually because they are jealous. These motives are natural enough, and their working out is told naturally and veridically, and although the passion is hardly of the type which attracts the modern novelist there is a very real and genuine passion. If there is no high tragedy there is plenty of quiet humour and subtle observation.

